# LINE-1 hypomethylation in gastric cancer, detected by bisulfite pyrosequencing, is associated with poor prognosis

**DOI:** 10.1007/s10120-012-0209-7

**Published:** 2012-11-21

**Authors:** Hironobu Shigaki, Yoshifumi Baba, Masayuki Watanabe, Asuka Murata, Shiro Iwagami, Keisuke Miyake, Takatsugu Ishimoto, Masaaki Iwatsuki, Hideo Baba

**Affiliations:** Department of Gastroenterological Surgery, Graduate School of Medical Science, Kumamoto University, 1-1-1 Honjo, Kumamoto, Kumamoto 860-8556 Japan

**Keywords:** LINE-1 elements, Gastric cancer, Methylation, Epigenetics, Prognosis

## Abstract

**Background:**

Genome-wide DNA hypomethylation plays an important role in genomic instability and carcinogenesis. DNA methylation in the long interspersed nucleotide element-1, L1 (LINE-1) repetitive element is a good indicator of the global DNA methylation level. In some types of human neoplasms, LINE-1 methylation level is attracting interest as a predictive marker for patient prognosis. However, the prognostic significance of LINE-1 hypomethylation in gastric cancer remains unclear.

**Methods:**

Using 203 resected gastric cancer specimens, we quantified LINE-1 methylation using bisulfite-pyrosequencing technology. A Cox proportional hazards model was used to calculate the hazard ratio (HR), adjusted for the clinical and pathological variables.

**Results:**

Gastric cancers showed significantly lower LINE-1 methylation levels compared to matched normal gastric mucosa (*p* < 0.0001; *n* = 74). Tumoral LINE-1 methylation range was 11.6–97.5 on a 0–100 scale (*n* = 203; mean 71.4, median 74.4, standard deviation 12.9). LINE-1 hypomethylation was significantly associated with shorter overall survival [log-rank *p* = 0.029; univariate HR 2.01, 95 % confidence interval (CI) 1.09–3.99, *p* = 0.023; stage-matched HR 1.88, 95 % CI 1.02–3.74, *p* = 0.041; multivariate HR 1.98, 95 % CI 1.04–4.04, *p* = 0.036]. No significant effect modification was observed by any of the covariates in survival analysis (all *p* interaction >0.25).

**Conclusions:**

LINE-1 hypomethylation in gastric cancer is associated with shorter survival, suggesting that it has potential for use as a prognostic biomarker.

## Introduction

Gastric cancer is a very common disease, the fourth most commonly diagnosed cancer and the second most common cause of cancer mortality globally [1]. Despite the developments in diagnosis and treatment technologies, the prognosis of gastric cancer patients remains poor, even for those who undergo complete resection of their carcinomas [2]. After the results of trastuzumab in patients with HER2-positive gastric cancer, there is increasing interest in the development of targeted therapies in this lethal disease [3]. Importantly, epigenetic changes, including alterations in DNA methylation, are reversible, and can thus be targets for therapy or chemoprevention [4–6]. In addition, the identification of new prognostic or predictive molecular markers for gastric cancer could improve the risk-adapted treatment strategies and help stratify patients in future clinical trials for drugs targeting these molecular changes.

DNA methylation is a fundamental epigenetic process that modulates gene expression. Cancer cells show two types of DNA methylation alterations: global DNA hypomethylation and site-specific CpG island promoter hypermethylation [7–9]. Global DNA hypomethylation plays a crucial role in genomic instability, leading to cancer development and progression [10–12]. Because LINE-1 or the L1 retrotransposon constitutes a substantial portion (approximately 17 %) of the human genome, LINE-1 methylation levels are regarded as a surrogate marker of global DNA methylation [13]. Although LINE-1 hypomethylation is strongly associated with a poor outcome in several types of human neoplasms [14–16], the influence of LINE-1 hypomethylation on the prognosis of gastric cancer patients remains unclear. Given the potential relationship between LINE-1 methylation level and genomic instability, we hypothesized that LINE-1 methylation level might mark an aggressive type of gastric cancer.

In this study, to test this hypothesis, we quantified LINE-1 methylation in 203 samples of resected gastric cancers utilizing a bisulfite-polymerase chain reaction (PCR)-pyrosequencing assay, and examined the prognostic significance of LINE-1 hypomethylation in gastric cancer. Our data suggest that LINE-1 hypomethylation can have a potential role as a prognostic biomarker.

## Materials and methods

### Study subjects

A total of 247 consecutive patients with gastric cancer who were undergoing resection at Kumamoto University Hospital between April 2005 and December 2009 were enrolled in this study. Nineteen patients were excluded for reasons of unavailability of adequate tissue samples. Because 22 patients received preoperative treatment, they were excluded from this study. Thus, we initially quantified LINE-1 methylation in 206 cancer specimens and obtained valid results in 203 (99 %) of the cases. Thus, a total of 203 gastric cancers were finally included in this study, and 74 cases were randomly chosen from these 203 cases to evaluate LINE-1 methylation level in normal matched mucosa. Patients were observed at 1- to 3-month intervals until death or 30 June 2011, whichever came first. Tumor staging followed the American Joint Committee on Cancer Staging Manual (7th edition) [17]. Overall survival was defined as the time between the date of the operation and the date of death. In our cohort, the 3-year overall survival rates of patients treated by gastrectomy were 91.9 % for stage I, 79.0 % for stage II, 56.8 % for stage III, and 19.5 % for stage IV. These rates are similar to those from the Japanese Gastric Cancer Association nationwide registry (94.1 % for stage I, 78.4 % for stage II, 53.2 % for stage III, and 22.4 % for stage IV), certainly supporting the absence of bias in our database. Written informed consent was obtained from each subject, and the study procedures were approved by the institutional review board. The term “prognostic marker” was used throughout this article according to the REMARK Guidelines [18].

### DNA extraction and sodium bisulfite treatment

Hematoxylin and eosin (H&E)-stained slides of the tumors were reviewed, and areas of tumors and histologically normal gastric mucosae adjacent to tumors were marked by one pathologist (Y.B.). H&E-stained tissue sections of the largest cross-sectional slice (depending on tissue and tumor size; on average, large tumor tissue 10 μm × 1 section) from each case were scraped off slides for DNA extraction. Genomic DNA was extracted from the tumor and normal epithelium. Genomic DNA was modified with sodium bisulfite using an EpiTect Bisulfite kit (Qiagen).

### Pyrosequencing to measure the LINE-1 methylation

**Fig. 1:**
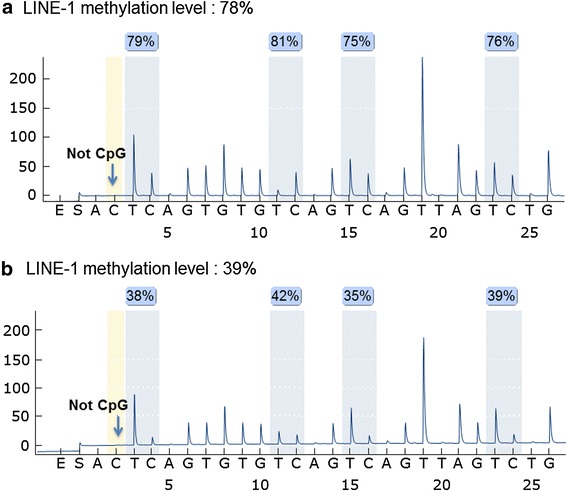
Pyrosequencing assay used to measure the long interspersed nucleotide element-1, L1 (LINE-1) methylation level. **a** A LINE-1 hypermethylated tumor (methylation level, 78 %). **b** A LINE-1 hypomethylated tumor (methylation level, 39 %). The percent (%) (*blue*) is the proportion of C at each CpG site after bisulfite conversion, and the methylation level of each CpG site was estimated by the proportion of C (%). The overall LINE-1 methylation level was calculated as the average of the proportions of C (%) at the 4 CpG sites. The first, third, and fourth CpG sites follow mononucleotide T repeats, resulting in higher T peaks than the second CpG site, and the proportion of C (%) has been adjusted accordingly. *Arrows* indicate no residual C at the non-CpG site, ensuring complete bisulfite conversion

PCR and subsequent pyrosequencing for LINE-1 were performed as previously described by Ogino et al., using the PyroMark kit (Qiagen) [14, 19, 20]. This assay amplifies a region of LINE-1 element (position 305–331 in accession no. X58075), which includes four CpG cites. The PCR conditions were 45 cycles of 95 °C for 20 s, 50 °C for 20 s, and 72 °C for 20 s, followed by 72 °C for 5 min. The biotinylated PCR product was purified and made single-stranded to act as a template in a pyrosequencing reaction, using the Pyrosequencing Vacuum Prep Tool (Qiagen). Pyrosequencing reactions were performed in the PyroMark Q24 System (Qiagen). The nucleotide dispensation order was ACT CAG TGT GTC AGT CAG TTA GTC TG. The non-CpG cytosine in LINE-1 repetitive sequences has been documented to be rarely methylated. Thus, complete conversion of cytosine at a non-CpG site ensured successful bisulfite conversion. The amount of C relative to the sum of the amounts of C and T at each CpG site was calculated as the percentage (i.e., 0–100). The average of the relative amounts of C in the 4 CpG sites was used as the overall LINE-1 methylation level in a given tumor (Fig. 1). In published literature, we have validated our LINE-1 methylation pyrosequencing assay; we have performed bisulfite conversion on five different DNA specimen aliquots and repeated PCR-pyrosequencing five times using four macro-dissected cancers. Bisulfite-to-bisulfite (between-bisulfite treatment) standard deviation (SD) ranged from 1.4 to 2.9 (median, 2.3), and run-to-run (between-PCR pyrosequencing run) SD ranged from 0.6 to 3.3 (median, 1.2) [21]. In this study, we used “LINE-1 methylation level” for LINE-1 methylation as a continuous variable and “LINE-1 hypomethylation” for LINE-1 methylation as a categorical variable (i.e., hypomethylation vs. hypermethylation).

### Statistical methods

For the statistical analyses, we used the JMP (Version 9; SAS Institute, Cary, NC, USA) and the SAS software programs (Version 9.1; SAS Institute). All *p* values were two sided. To compare the means, we performed the *t* test assuming unequal variances. For the survival analysis, the Kaplan–Meier method was used to assess the survival time distribution, and the log-rank test was used. To assess the independent effect of the LINE-1 methylation level on mortality, the tumor stage (I, II, III + IV) was used as a stratifying (matching) variable in Cox models using the “strata” option in the SAS “procphreg” command to avoid residual confounding and overfitting. We constructed a multivariate, stage-stratified Cox proportional hazard model to compute a hazard ratio (HR) according to LINE-1 methylation status, containing sex (male vs. female), age at surgery (continuous variable), tumor location (lower vs. middle or upper), and histological type (intestinal vs. diffuse). A backward stepwise elimination with a threshold of *p* = 0.20 was used to select variables in the final model. We initially performed the Cox regression analysis with LINE-1 methylation as a continuous variable and then performed the Cox regression analysis with LINE-1 methylation as a categorical variable. An interaction was assessed by including the cross product of the LINE-1 variable and another variable of interest in a multivariate Cox model; thereafter, the Wald test was performed.

## Results

### LINE-1 methylation in gastric cancer and matched noncancerous mucosa

**Fig. 2:**
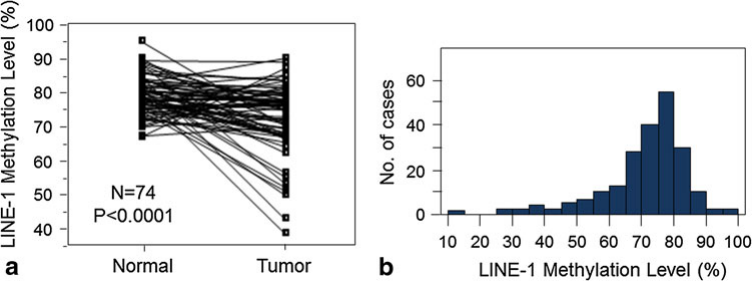
**a** LINE-1 methylation levels in 74 gastric cancer and matched normal mucosa specimens. The cancer tissues showed significantly lower levels of methylation than matched normal mucosa (*p* < 0.0001 by paired *t* test). **b** Distribution of LINE-1 methylation levels in 203 gastric cancers

We first examined LINE-1 methylation level in 74 gastric cancer tissues and matched noncancerous mucosa samples. The cancer tissues exhibited significantly lower levels of LINE-1 methylation [median 74.9, mean 72.3, SD 10.1 (all in 0–100 scale)] than matched noncancerous mucosa (median 79.4, mean 79.2, SD 5.6) (*p* < 0.0001 by the paired *t* test) (Fig. 2a).

### Evaluation of the association of LINE-1 methylation level and clinical and pathological variables

**Fig. 3:**
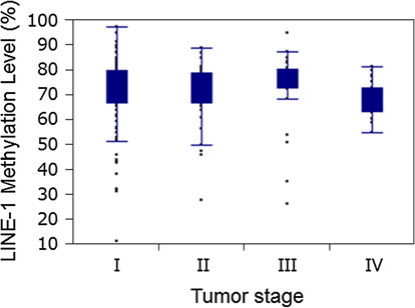
Analysis with LINE-1 methylation as a continuous variable showed no significant relationship between LINE-1 methylation level and tumor stage (*p* = 0.64)

Next, we quantified the LINE-1 methylation in 206 cancer specimens and obtained valid results in 203 (99 %) of cases. LINE-1 methylation levels in the 203 cancers (Fig. 2b) were approximately normally distributed: mean 71.4, median 74.4, SD 12.9, range 11.6–97.5; inter-tertile range 70.0–77.4 (all in 0–100 scale). The LINE-1 methylation level was then divided into tertiles [Ter1 (77.4–97.5, *n* = 68), Ter2 (70.1–77.3, *n* = 66), Ter3 (11.6–70.0, *n* = 69)] for further analyses. We found that the LINE-1 methylation level was associated with tumor stage (*p* = 0.039; Table 1). However, in the analysis with LINE-1 methylation as a continuous variable, there was no significant relationship between LINE-1 methylation level and tumor stage (*p* = 0.64, Fig. 3). LINE-1 methylation was not significantly associated with other clinical or pathological variables.

**Table 1:** Long interspersed nucleotide element-1, L1 (LINE-1) methylation in gastric cancer specimens and association with clinical and tumor features

Clinical or pathological feature	Total (*n*)	LINE-1 methylation (tertile)			*p* value
		Ter1 (77.4–97.5)	Ter2 (70.1–77.3)	Ter3 (11.6–70.0)	
All cases	203	68	66	69	
Mean age (years) ± SD	70.0 ± 10.4	69.2 ± 10.2	69.3 ± 9.5	71.5 ± 11.4	0.35
Sex					
Female	55 (27 %)	18 (26 %)	20 (30 %)	17 (25 %)	0.75
Male	148 (73 %)	50 (74 %)	46 (70 %)	52 (75 %)	
Year of diagnosis					
2000–2005	77 (38 %)	24 (35 %)	28 (42 %)	25 (36 %)	0.66
2006–2009	126 (62 %)	44 (65 %)	38 (58 %)	44 (64 %)	
Tumor location					
Lower	72 (35 %)	30 (44 %)	23 (35 %)	19 (28 %)	0.09
Middle	66 (33 %)	19 (28 %)	17 (26 %)	30 (44 %)	
Upper	65 (32 %)	19 (28 %)	26 (39 %)	20 (29 %)	
T classification					
T1a + b	100 (49 %)	32 (47 %)	36 (55 %)	32 (46 %)	0.43
T2	24 (12 %)	9 (13 %)	4 (6.1 %)	11 (16 %)	
T3	48 (24 %)	17 (25 %)	18 (27 %)	13 (19 %)	
T4a + b	31 (15 %)	10 (15 %)	8 (12 %)	13 (19 %)	
*N* classification					
N0	130 (64 %)	41 (60 %)	44 (67 %)	45 (65 %)	0.91
N1	29 (14 %)	13 (19 %)	8 (12 %)	8 (12 %)	
N2	18 (9 %)	6 (9 %)	6 (9 %)	6 (18 %)	
N3	26 (13 %)	8 (12 %)	8 (12 %)	10 (15 %)	
Stage					
I (IA, IB)	111 (55 %)	39 (57 %)	36 (55 %)	36 (52 %)	0.039
II (IIA, IIB)	40 (20 %)	13 (19 %)	16 (24 %)	11 (16 %)	
III (IIIA, IIIB, IIIC)	25 (12 %)	11 (16 %)	9 (14 %)	5 (7.3 %)	
IV	27 (13 %)	5 (7.4 %)	5 (7.6 %)	17 (25 %)	
Histological type					
Intestinal	130 (64 %)	43 (63 %)	47 (71 %)	40 (58 %)	0.27
Diffuse	73 (36 %)	25 (37 %)	19 (29 %)	29 (42 %)	

Percent (%) indicates the proportion of cases with a specific clinical or pathological feature among each tertile group (Ter1, Ter2, or Ter3)

### LINE-1 hypomethylation and patient survival

**Fig. 4:**
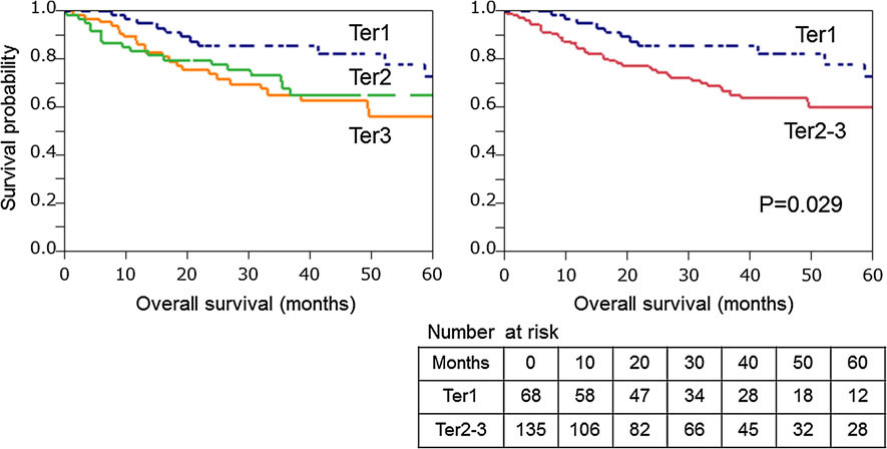
Kaplan–Meier curves for overall survival according to tertiles (Ter1–3) of LINE-1 methylation in gastric cancer. In panels on the *right*, Ter2–3 represents the hypomethylated group and Ter1 represents the hypermethylated group

During the follow-up of the 203 patients, there were a total of 56 deaths. The median follow-up time for censused patients was 2.9 years. The primary statistical survival analysis was the Cox regression test with LINE-1 methylation as a continuous variable. LINE-1 hypomethylation was associated with a statistically significant increase in overall survival rate (univariate analysis *p* = 0.014). The univariate hazard ratio for overall survival rate associated with a 20 % decrease in LINE-1 methylation was 1.96 [95 % confidence interval (CI) = 1.33–2.87]. We also performed analyses using categorical variables (i.e., tertile). In a univariate Cox regression analysis, compared to first tertile (Ter1) cases, the third tertile (Ter3) cases experienced a significantly lower overall survival rate (*p* = 0.017, HR 2.24, 95 % CI 1.15–4.63). The second tertile (Ter2) cases experienced a slightly, but not significantly, lower overall survival rate compared to Ter1 cases (*p* = 0.12, HR 1.76, 95 %CI 0.85–3.74) (Table 2; Fig. 4). Based on these results, we made a dichotomous LINE-1 methylation variable (i.e., hypomethylation vs. hypermethylation), defining Ter1 as the “hypermethylated group” and combining Ter2 and Ter3 into the “hypomethylated group.” Thus, in this study, “LINE-1 hypomethylation” was defined as “≤77.3 %” and “LINE-1 hypermethylation” was defined as “≥77.4 %.”

**Table 2:** Association of LINE-1 methylation status in gastric cancer with patient survival

LINE-1 methylation level (tertile)	Total (*n*)	Overall survival		
		Univariate HR (95 % CI)	Stage-matched HR (95 % CI)	Multivariate stage-matched HR (95 % CI)
Ter1 (≥77.4)	68	1 (referent)	1 (referent)	1 (referent)
Ter2 (70.1–77.3)	66	1.76 (0.85–3.74)	1.89 (0.92–4.02)	2.01 (0.96–4.36)
Ter3 (≤70.0)	69	2.24 (1.15–4.63)	1.88 (0.96–3.90)	1.96 (0.95–4.21)
Ter1 (≥77.4)	68	1 (referent)	1 (referent)	1 (referent)
Ter2–3 (<77.3)	135	2.01 (1.09–3.99)	1.88 (1.02–3.74)	1.98 (1.04–4.04)
*p* value		0.023	0.041	0.036

*CI* confidence interval, *HR* hazard ratio

In the Kaplan–Meier analysis, LINE-1 hypomethylators (i.e., Ter2 and Ter3 cases) experienced significantly shorter overall survival (log rank *p* = 0.029) than those with hypermethylation (Fig. 4). In the univariate Cox regression analysis, compared to LINE-1 hypermethylated cases, LINE-1 hypomethylators experienced a significantly lower overall survival rate (HR 2.01, 95 % CI 1.09–3.99, *p* = 0.023) (Table 2). In the multivariate Cox model adjusted for the clinical and pathological features, LINE-1 hypomethylation was found to be associated with a significantly lower overall survival rate (multivariate HR 1.98, 95 % CI 1.04–4.04, *p* = 0.036). Another independent prognostic factor was histological diffuse type (multivariate HR 1.91, 95 % CI 1.09–3.29, *p* = 0.023), whereas neither sex, age, nor tumor location was significantly associated with overall survival rate.

### Interaction between LINE-1 hypomethylation and other variables in the survival analyses

We also examined whether the influence of LINE-1 hypomethylation on the overall survival was modified by any of the clinical and pathological variables. We did not observe a significant effect of modification by any of the covariates in survival analysis (all *p* interaction >0.25). Notably, there was no significant interaction between LINE-1 methylation and tumor stage (*p* interaction = 0.68 for stage I, II vs. III, IV; *p* interaction = 0.97 for stage I vs. II–IV).

## Discussion

In this study, we examined the prognostic impact of LINE-1 hypomethylation among 203 patients with resected gastric cancer. Because LINE-1 constitutes a substantial portion of the human genome, the methylation status of LINE-1 reflects global DNA methylation level [13]. We have found that LINE-1 hypomethylation (i.e., global DNA hypomethylation) in gastric cancer is associated with a poor prognosis, suggesting that LINE-1 hypomethylation may be a biomarker that can be used to identify patients who will experience an inferior outcome.

Although the prognostic factors in gastric cancer have been extensively studied [22–25], little is known regarding the prognostic value of global DNA hypomethylation. The relationship between LINE-1 hypomethylation and poor prognosis has been reported in several types of human neoplasms (e.g., prostate [26], colon [14], and ovarian [16] cancers and in chronic myeloid leukemia [27]). Our current finding in gastric cancer is in agreement with these results. On the other hand, a study of cutaneous melanoma has demonstrated that LINE-1 hypomethylation is associated with a favorable outcome [28], which did not agree with our current finding. This discrepancy might result from differences in the tumor histological type. Our data certainly support a potential role for LINE-1 hypomethylation as a prognostic biomarker for gastric cancer.

Cancer cells exhibit two types of DNA methylation alterations: global DNA hypomethylation and site-specific CpG island promoter hypermethylation [29]. It is well established that tumor suppressor genes can be silenced through promoter CpG island methylation during carcinogenesis [5, 30]. In gastric cancer, a large number of genes (e.g., *CDKN2A*, *CDK2AP2*, *CDH1*, *MGMT*, *RASSF1*, *RUNX3*, and *DLC1*) have been shown to be suppressed by CpG island hypermethylation [31]. Of these genes, promoter hypermethylation of *CDH1* [32] and *MGMT* [33, 34] was associated with worse outcomes after surgery for gastric cancer. In contrast, the prognostic significance of global DNA hypomethylation is still unknown. To the best of our knowledge, this is the first study evaluating the relationship between LINE-1 methylation level and patient outcome in gastric cancer.

Accumulating evidence supports a crucial role of global DNA hypomethylation in tumor initiation and development: one study with a large sample collection of chronic gastritis, intestinal metaplasia, gastric adenoma, and gastric cancer demonstrated that aberrant DNA methylation occurred in early stages and tended to accumulate along the multistep gastric carcinogenesis [35]. In some types of human cancers including gastric cancer, global genomic hypomethylation has been found in the premalignant stages [36]. Nonetheless, whether global DNA hypomethylation influences cancer progression to a more advanced stage has remained uncertain. Our current finding on the relationship between LINE-1 hypomethylation and poor prognosis may support that global DNA methylation may contribute to not only initiation but also to progression of the gastric tumor.

The mechanism by which global DNA hypomethylation may confer a poor prognosis remains to be fully explored. First, genome-wide DNA hypomethylation has been shown to be associated with genomic instability [10–12, 37], which might confer a poor prognosis. Second, the transcriptional dysregulation might be another possible mechanism, and activation of proto-oncogenes, transposable elements, or endogenous retroviruses might affect the tumor aggressiveness. Third, in addition to its role as a surrogate marker for global DNA methylation, the LINE-1 methylation status by itself likely has biological effects, because retrotransposons, such as LINE-1 elements, can provide alternative promoters [38], and contribute to noncoding RNA expression, which regulates the functions of a number of genes [39, 40]. Further studies are necessary to validate our findings, as well as to elucidate mechanism(s) by which LINE-1 hypomethylation affects tumor malignant behavior.

There are limitations in this study. Our cohort included relatively large numbers of patients (*n* = 203), but the validation cohort was missing. Our findings need to be validated in an independent dataset. In addition, epidemiological data (e.g., smoking history, alcohol drinking history, *Helicobacter pylori* infection) were limited.

In summary, the current study suggests that genome-wide DNA hypomethylation, as measured in LINE-1, is independently associated with poor survival among patients with gastric cancer. Future studies are needed to confirm this association, as well as to examine the potential mechanism by which genome-wide DNA hypomethylation affects tumor behavior or progression.
